# Mattei Remedies and Cure for Drunkenness

**Published:** 1893-12-09

**Authors:** 


### EDITOR'S LETTER-BOX.

[Our correspondents are reminded that proxility is a great bar to publication, and that brevity of style and conciseness of statement greatly facilitate early insertion.]

### MATTEI REMEDIES AND CURE FOR DRUNKENNESS.

Dr. Hugh Woods writes: I am investigating the Mattei remedies for cancer, and the cures for drunkenness emanating from the same source—namely, Mr. Gliddon, 18, Pall Mall East, S.W. I shall, therefore, be very greatly obliged to any of your readers who can furnish me with the names and addresses of persons who have taken these remedies, and any details as to such cases. In the interests of medical science and the medical profession, it is of the utmost importance that the claims of these much-advertised cures for various diseases should be thoroughly sifted. Communications should be addressed Dr. Hugh Woods, 11, Archway Road, Highgate, London, N.

